# Dynamic Range Expansion of the C-Reactive Protein Quantification with a Tandem Giant Magnetoresistance Biosensor

**DOI:** 10.1021/acsomega.1c01603

**Published:** 2021-05-07

**Authors:** Fanda Meng, Lei Zhang, Weisong Huo, Jie Lian, Aldo Jesorka, Xizeng Shi, Yunhua Gao

**Affiliations:** † Department of Clinical Laboratory Medicine, The First Affiliated Hospital of Shandong First Medical University & Shandong Provincial Qianfoshan Hospital, Shandong Medicine and Health Key Laboratory of Laboratory Medicine, Jinan 250014, China; ‡ School of Basic Medicine, Shandong First Medical University & Shandong Academy of Medical Sciences, Jinan 250062, China; § Department of Chemistry and Chemical Engineering, Chalmers University of Technology, Gothenburg SE-412 96, Sweden; ∥ Key Laboratory of Photochemical Conversion and Optoelectronic Materials, Technical Institute of Physics and Chemistry, Chinese Academy of Sciences, Beijing 100190, China; ⊥ University of Chinese Academy of Sciences, Beijing 100149, China; # Dongguan Bosh Biotechnologies, Ltd., Guangdong 523808, China; ∇ College of Criminal Investigation, People’s Public Security University of China, Beijing 100038, China

## Abstract

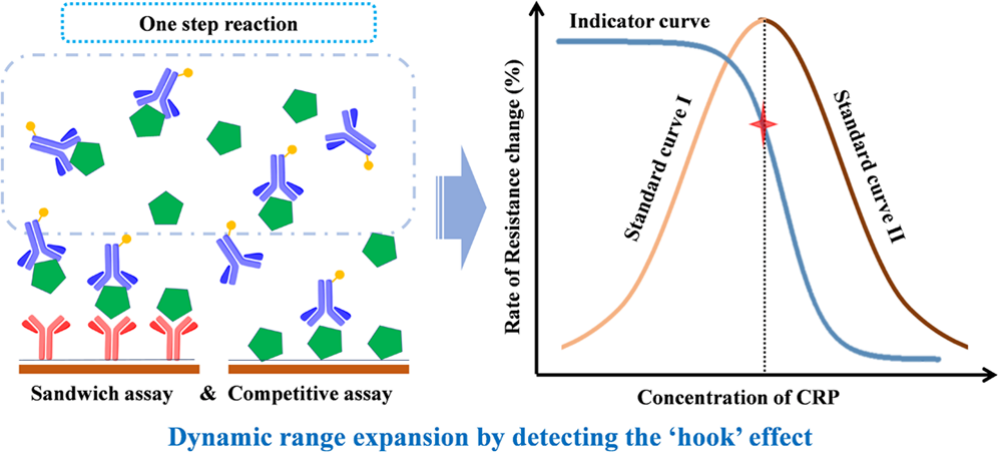

In this study, we report a convenient analytical method for a full-range quantification of the C-reactive protein (CRP), a blood biomarker of infection and cardiovascular events. We determine CRP over the entire diagnostically relevant concentration range in undiluted human blood serum in a single test, using a tandem giant magnetoresistance (GMR) sensor. The tandem principle combines a sandwich assay and a competitive assay, which allows for the discrimination of the concentration values resulting from the multivalued dose–response curve (“Hook” effect), which characterizes the one-step sandwich assay at high CRP concentrations. The sensor covers a linear detection range for CRP concentration from 3 ng/mL to 350 μg/mL, the detection limit (s/n = 3) is 1 ng/mL. The prominent features of the chip-based method are its expanded dynamic range and low sample volume (50 μL), and the need for a short measurement time of 15 min. These figures of merit, in addition to the low detection limit equal to the established assay instrumentation, make it a viable candidate for use in point-of-care diagnostics.

## 1 Introduction

CRP, mainly synthesized in the liver upon an acute inflammatory stimulus, has been found to be a potent biomarker of infection and pathological cardiovascular events. The levels of C-reactive protein (CRP) are increased in many disorders, and it is regarded as a very good predictor of disease state, particularly cardiac risk stratification.^1−3^ If the levels of CRP in serum are below 1.0 μg/mL, the risk of cardiovascular diseases is considered low; levels between 1 and 3 μg/mL indicate a moderate risk and levels greater than 3 μg/mL are considered a significant indicator for chronic cardiovascular disease, including acute coronary syndromes.^4,5^ Elevated levels between 10 and 50 μg/mL can also be detected in viral infections and late pregnancy, and levels between 50 and 200 μg/mL are typically associated with bacterial infections and active inflammation.^2,6^ Values >200 μg/mL are comparatively rare, which indicate severe health issues of the affected individuals. Bacterial infections account for the majority of instances of extreme CRP elevation, and mortality is high.^7^ To design a universal CRP assay that is useful in diverse disease contexts, it should span the whole concentration range, which characterizes the clinically relevant levels of CRP, i.e., from <1.0 to >200 μg/mL.

The most commonly utilized analytical techniques currently employed for the quantification of CRP include the enzyme-linked immunosorbent assay (ELISA),^8^ biosensors,^9−13^ and lab-on-a-chip devices,^14,15^ with good figures of merit including low detection limits reaching picomolar concentrations. The methods derived from these techniques typically detect CRP in diluted human whole blood and serum. Moreover, approved clinical detection methods of CRP generally require expensive analytical equipment, elaborate and time-consuming experimental procedures, and trained personnel, which also make automated and high-throughput analyses rather difficult.

To develop an alternative analytical biomarker test for application in clinical test practice, one or more significant benefits are required. Such benefits would include the direct use of undiluted blood serum and direct access to the clinically relevant concentration range of CRP in a single analysis. Dilution is generally the preferred method to circumvent the “Hook” effect, but it would prevent the detection of low-abundance species. As CRP is typically determined in conjunction with low-abundance infection biomarkers, the dilution of the serum poses a serious practical problem. A chip format using a sensor array for simultaneous analysis of several biomarker species with individual dynamic ranges would not only be capable of solving this problem but also be a promising prerequisite for automated and rapid analysis.

In our work toward such an improvement, we used a fully integrated point-of-care testing (POCT) platform based upon a giant magnetic resistance (GMR) biosensor array, combined with microfluidic sample handling circuitry. The platform was modified to implement a new sensor configuration, designed to expand the dynamic range of CRP sensing. This allows for applying undiluted samples, which not only simplifies the existing measurement procedure but also opens pathways toward direct and simultaneous multianalyte quantification of both high- and low-abundance biomarkers.

In the setup, two individual sensors located on a chip array are differentially coated to become the foundation for complementary assay formats. On one sensor surface, a capture antibody is immobilized for a one-step sandwich assay, which uses a matched antibody pair: an immobilized capture antibody and a detection antibody mixed with the sample, both with affinity to CRP. On the other sensor, the surface features immobilized antigens for a competitive assay, which binds to the detection antibody in competition to CRP present in the sample. This tandem arrangement is capable of a single-run detection of CRP in concentration ranges typical for a variety of medical conditions.

The one-step sandwich assay is currently the most used format in clinical and point-of-care immunoassays due to its high speed. The major drawback is the accessible concentration range, which is commonly limited by the so-called “Hook” effect, i.e., a multivalued dose–response relationship.^16,17^ The “Hook” effect, only observed in one-step assays, is caused by the excess of analyte and prevents simultaneous binding of solid-phase and liquid-phase antibodies. Even though the use of excess antibody postpones the “Hook” effect to a certain extent in theory, it also greatly increases the cost of immunoassay.

In the GMR sensor array, the sandwich assay on sensor I will determine the full dose–response relationship, which results in the conventionally undesirable “Hook”-shaped curve as the concentration increases toward the upper limit, while the competitive assay on sensor II detects at the peak point of the “Hook” curve, which indicates whether the “Hook” effect occurred or not in the sandwich assay. The combination of both assays is used to determine the overall signal, which allows for a full-range measurement. Moreover, since this arrangement requires only 2 out of the 12 on-chip sensors per analysis, the remaining spare channels can be utilized for complementary assays targeting additional biomarkers.

## 2 Materials and Methods

### 2.1 Regents and Materials

Na_2_HPO_4_, NaHCO_3_, KCl, Na_2_CO_3_, NaH_2_PO_4_, NaCl, and bovine serum albumin (BSA) were purchased from Sigma Aldrich (Merck). NHS-Biotin was purchased from Thermo Fisher Scientific, Inc. Streptavidin-conjugated magnetic particles (MNP, 100 nm) were purchased from Ademtech (France). Tween-20 was purchased from AMRESCO. A laboratory quantity of polystyrene-grafted maleic anhydride (PS-g-MA, graft ratio 17%) was provided free of charge by Longjia Plastics Fabrication (Jilin, China). Heterophilic blocking reagent (HBR1) was obtained from Scantibodies Laboratory, Inc. and heterophilic immunoglobulin elimination reagent (Fapon Block: HIER-E-010) from Fapon Biotech Inc. (China). All commercial reagents were of analytical grade.

Antihuman CRP antibodies (Capture antibody:MCP01, Detection antibody:MCP02) and CRP antigen were purchased from Hangzhou Yibaixin Biotechnology Co., Ltd. (China). Undiluted clinical serum samples were received as a donation from the Zhujiang Hospital of Southern Medical University (China).

The GMR immunoassay analyzer (Bosh M16) and the polymer assay cartridge were manufactured by Dongguan Bosh Biotechnologies, Ltd. (China). The use of this device has been reported elsewhere.^18,19^

Carbonate buffer (CB, 0.1 M, pH 9.6) was used for the immobilization of the capture antibody and capture antigen. CBTB, used to prepare and redissolve the MNP solution, was CB buffer spiked with 0.05% Tween-20 and 10% BSA. Phosphate-buffered saline (PBS, 10 mM, pH 7.4) was created by mixing NaH_2_PO_4_ and NaH_2_PO_4_. PBSB, composed of 10 mM PBS spiked with 10% BSA, was used to prepare the labeling antibody solution. PBST, prepared by PBS spiked with 0.1% Tween-20, was used as a washing buffer. The MNP solution, used in the assay, was washed three times and diluted 1:10 by CBTB. The detection antibody was biotinylated using NHS-biotin^20^ and diluted by PBSB. Fetal bovine serum (Sigma Aldrich) was used for the preparation of the he CRP standard solutions.

### 2.2 GMR Chip Preparation

The GMR measurement is based on the Wheatstone bridge principle. The resistance change in the sensor is compensated, and the ratio (*R* – *R*_0_)/R_0_ × 100 is stated as the rate of resistance change, where *R* is the resistance of the GMR sensor after immunoreaction in the specific magnetic field and *R*_0_ is the resistance of blank sensor without the magnetic field.

**Figure 1:**
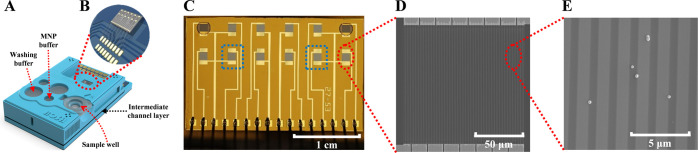
POCT assay cartridge used in the study. A scheme of the channel architecture has been published elsewhere.^19^ (A) Schematic view of the full microfluidic sample handling device with on-chip sample wells. (B) GMR sensor chip contained in the cartridge, featuring 12 individual sensors. (C–E) Successive magnifications of a single GMR sensor array chip, obtained by optical microscopy and scanning electron microscopy (SEM). The two selected sensor units used in tandem are marked by a blue square in (C).

The reagents needed in the array were supplied to the corresponding cartridge wells (sample, washing buffer, MNP buffer) (Figure 1A). The GMR chip^18^ (2.66 mm × 1.62 mm) containing 12 individual GMR sensors (120 μm × 120 μm each) (Figure 1B) was surface-modified and combined with the cartridge body (top structure with wells) and an intermediate channel layer (microfluidic circuitry) prior to use. A beneficial feature of the GMR sensor array is the ability to perform parallel analyses, which is useful for the tandem assay, but also for performing repeat measurements and simultaneous analyses of additional biomarkers. In this research, two individual sensors from this array (Figure 1C–E) were modified as a sandwich array sensor and a competitive array sensor. Note that the top corners are marked, which is necessary for the nanoplotter to determine the positions of the sensors. These points are optically autorecognized by the plotter.

**Figure 2:**
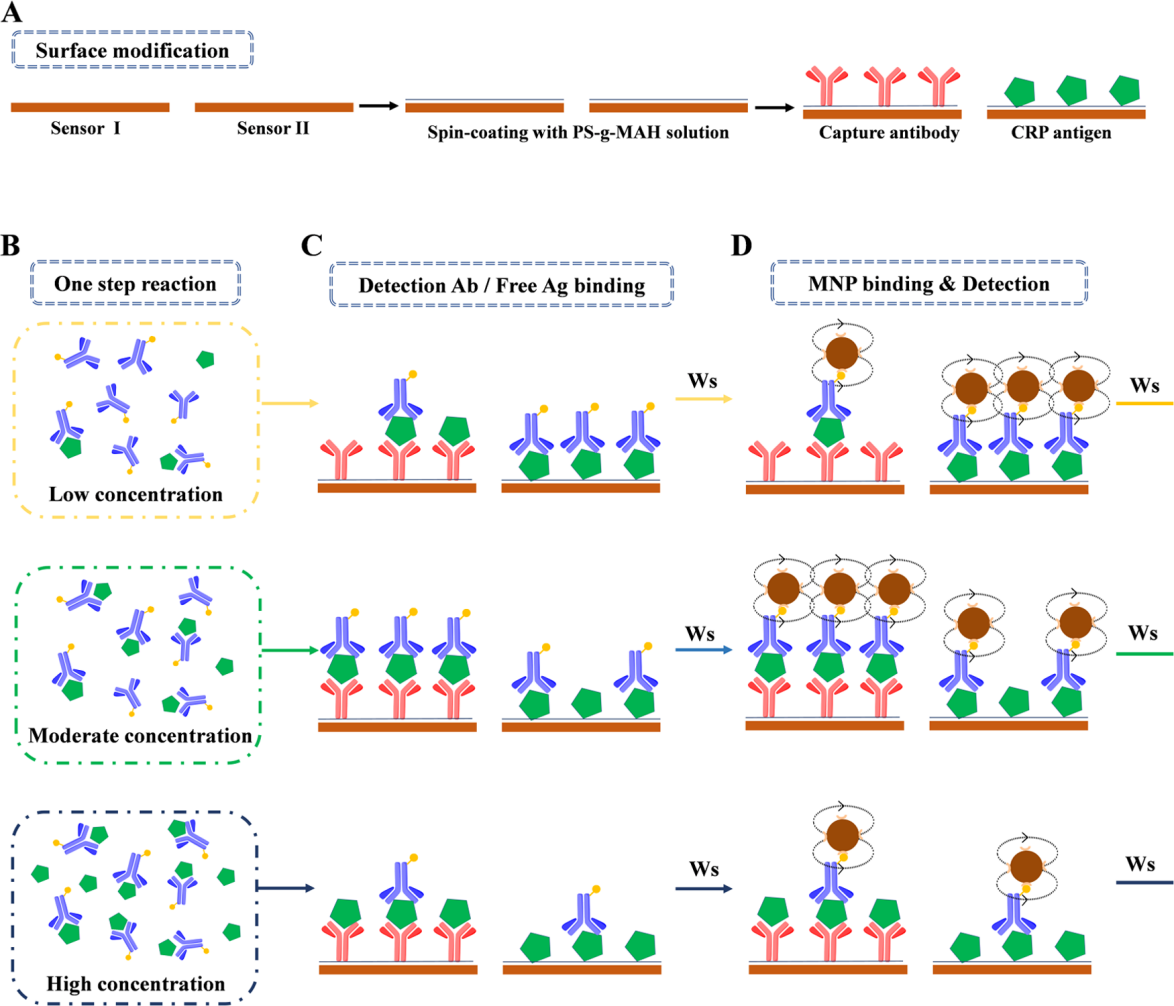
Assay protocol for detecting the “Hook” effect to quantify CRP. (A) Surface modification. Sensor I: sandwich array sensor. Sensor II: indirect competitive assay sensor. (B) One-step antibody–antigen reaction after sample loading. (C) Biotin-labeled detection antibody or free antigen binding. (D) Magnetic particle binding and detection. Ws: washing step.

The GMR chip surface was coated with PS-g-MAH in a 1% (w/v) solution in toluene via spin coating (EZ-4 spin coater, Zhengzhou CY Scientific Instrument Co., Ltd., I: 800 rpm, 30 s, II: 2000 rpm, 60 s) and annealed at 70 °C for 10 min in a laboratory convection oven (ZD-85, Jintan Jincheng Guosheng Experimental Instrument Co. Ltd., China) (Figure 2A). The CRP capture antibody and antigen solutions were deposited onto the sensors using an NP 2.1 Nano-Plotter (GeSiM, Germany) and thereafter incubated for 30 min at 37 °C and 70% humidity. The antibody (sensor I) and antigen (sensor II) molecules are chemically conjugated to the MAH functionalities on the surface.^21^ The unreacted PS-g-MAH will be directly deactivated upon incubation at 37 °C and high humidity, so a blocking step is unnecessary. The detection antibody solution (10 μL) and the MNP solution (10 μL) were pipetted to the designated reservoirs of the intermediate channel layer and freeze-dried. Subsequently, the cartridge was assembled. Since some proteins in human serum may undergo nonspecific binding, we have solved this problem by preadding commercial blockers (HBR1: 200 μg/mL × 5 μL & Fapon Blocker: 250 μg/mL × 5 μL) to the sample well, followed by freeze-drying.

### 2.3 Immunoassay Procedure

For the purpose of expanding the measurement range, covering the concentration range relevant for clinical analysis, we combined a CRP antigen-modified sensor as a competitive array sensor with a sensor modified for a sandwich assay (Figure 2A–D). In the presence of low concentrations of antigen, the sandwich array sensor I, which uses a one-step antibody–antigen binding reaction, generates a response signal that increases with concentration. In contrast, in the presence of high concentrations of antigen, the sandwich array sensor signal decreases with the CRP concentration. Sensor II, modified for the competitive immunoassay, is utilized to monitor at which concentration the “Hook” effect occurs. The two signals are combined and processed to establish a unique dose–response relationship. The assay protocol (Figure 2A–D) comprises the first step of an immunoreaction of (sample or standard) antigen with detection antibodies (Figure 2B). On the surface of sensor I, the antigen–antibody pair attaches to the capture antibodies, forming the immunoassay sandwich (37 °C, 7 min; Figure 2C). Simultaneously, excess detection antibody binds to the antigen on the surface of sensor II (Figure 2C). Thereafter, the binding to avidin-coated magnetic nanoparticles occurs (6 min at 37 °C; Figure 2D). Washing steps (flow rate 50 μL/min, 1 min at 37 °C) are applied before and after the MNP binding step. The captured MNP (Figure 2D) are detected in the final step by the GMR sensors. Their output signals are processed, and calibration curves as well as the CRP concentration in the sample are obtained. The total analysis time is 15 min.

### 2.4 Data Processing

For the creation of the calibration curves, four reference measurements were performed for each concentration point, and means/standard deviations were calculated. Ninety-one patient samples were analyzed in total; one measurement was performed per patient sample.

**Figure 3:**
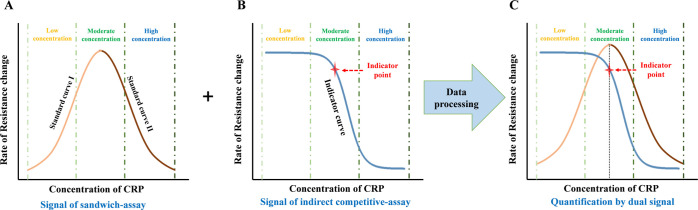
Principle of data processing. (A) Calibration curve obtained with external standards from sensor I, displaying the “Hook” effect (multivalued dose–response curve). (B) Indicator curve from sensor II. The measured signal intensity decreases with analyte concentration (competitive assay). The red star denotes the indicator point assigned for determining whether the “Hook” effect has occurred or not. (C) Superposition of the two calibration curves for the determination of the position of the indicator point.

By means of the sensor II signal, we detect the turning point in the “Hook”-shaped dose–response curve to discriminate between the low- and high-concentration situations (Figure 3). Standard curve I was used to quantify the concentration of CRP before the “Hook” effect occurs, while standard curve II was used to quantify the concentration of CRP after the peak point of the dose–response curve (Figure 3A). Using the established standard curves, we first located an indicator point according to the signal recorded with sensor II (Figure 3B). The indicator point is chosen by the superposition of both calibration curves (Figure 3C). The full indicator curve is not used for quantification in the usual manner of external calibration, only the indicator point is used. If the measured value is higher than the value of the indicator point, the signal from sensor I is used with standard curve I (Figure 3A, orange line). In the other case with the measured value less than the value of the indicator point, the signal from sensor II is used with standard curve II (Figure 3A, brown line).

### 2.5 Fitting Software and Algorithm

All standard curves were fitted by the four-parameter logistic model (*y* = *A*_2_ + (*A*_1_ – *A*_2_)/(1 + (*x*/*x*_0_)*^p^*)),^22^ using Origin 9.1 software.

## 3 Results and Discussion

To establish optimal values for all assay components as well as experimental conditions, (a) the concentrations of capture and detection antibody and (b) the amount of surface-immobilized antigen (sensor II) were optimized first. Thereafter, the reaction times of both integrated assays were optimized individually. Calibration curves for sensor I (21 external standards with *n* = 4) and sensor II (21 external standards with *n* = 4) were recorded and fitted to the selected model. Afterward, 91-patient samples were measured (*n* = 1) and validated with an equal number of samples on Roche Cobas C501.

### 3.1 Optimization of Detection of CRP

#### 3.1.1 Concentration of Antibody and Antigen

**Figure 4:**
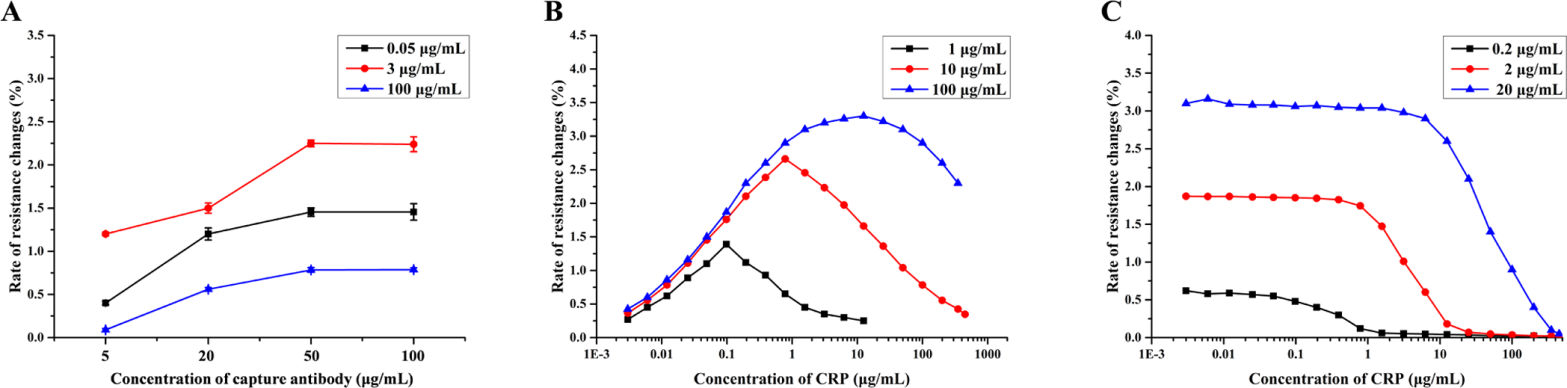
Optimization of the sensor signals. (A) Dependence of the signal magnitude (sensor I) on the concentration of the capture antibody (*n* = 4). The inset refers to the analyte (CRP) concentrations used to detect the point of saturation over the entire concentration range. (B) Dependence of the signal magnitude (sensor I) on the concentration of the detection antibody (*n* = 1). The inset refers to the different concentrations of detection antibody used to determine a hook-shaped curve that covers the entire concentration range of interest. (C) Dependence of the signal magnitude (sensor II) on the concentration of surface-immobilized antigen (*n* = 1). The inset refers to the concentrations of the capture antigen solution used to prepare the sensor surface.

The influence of the capture antibody concentration on the immune reaction of a sandwich assay was determined in the presence of 0.05, 3, and 100 μg/mL CRP (Figure 4A). The rate of resistance changes saturated as the concentration of the capture antibody exceeded 50 μg/mL. Accordingly, 50 μg/mL capture antibody was used in the assay.

The influence of detection antibody concentration on the sensor I signal (sandwich assay) was investigated with respect to the detection antibody concentration (1, 10, and 100 μg/mL; Figure 4B). Since both sensors are subjected to the same detection antibody solution, they cannot be individually optimized. The determination of the inversion point in the calibration curve is fundamental; therefore, the detection antibody concentration is optimized for sensor I. The optimal relationship that covers the full clinical range while requiring the lowest amount of detection antibody was found at a concentration of 10 μg/mL. In principle, as the blue line in Figure 4B suggests, up to a CRP concentration of 10 μg/mL, the assay could be run using a single sensor. However, the cost of the detection antibody decides the overall cost of a single assay so that there is no apparent benefit. Moreover, at very high CRP concentrations, the sensor response intensity is low, close to the limit of quantification (LOQ). To further optimize the assay for analytical situations specifically at the high end of the concentration range, a detection antibody concentration somewhere in between 10 and 100 μg/mL could be applied. Some mechanistic aspects concerning the appearance of the multivalued calibration curves in dependence on detection antibody concentration were reported earlier by Fernando et al.^16^

The optimal concentration of the CRP capture antigen for the signal intensity arising from the immune reaction of the competitive assay (sensor II), which matches the response of sensor I, was determined as 2 μg/mL (Figure 4C). To further optimize the assay for analytical situations that give a better indicator, a capture antigen concentration somewhere between 0.2 and 2 μg/mL could be applied.

One additional factor that would possibly benefit from optimization is the quality of the functionalized sensor surfaces, which depends on a variety of process parameters. The concentration of the PS-g-MAH solution, solvent, parameters of spin coating, and drying conditions could have an influence on the final functionalization and can be made the subject of optimization. Currently, we do not have established information on surface-related parameters, such as the number and density of antibodies, their distribution, or molecular conformation, and orientation or shape on the sensor surface. These aspects are still under investigation.

#### 3.1.2 Reaction Time

**Figure 5:**
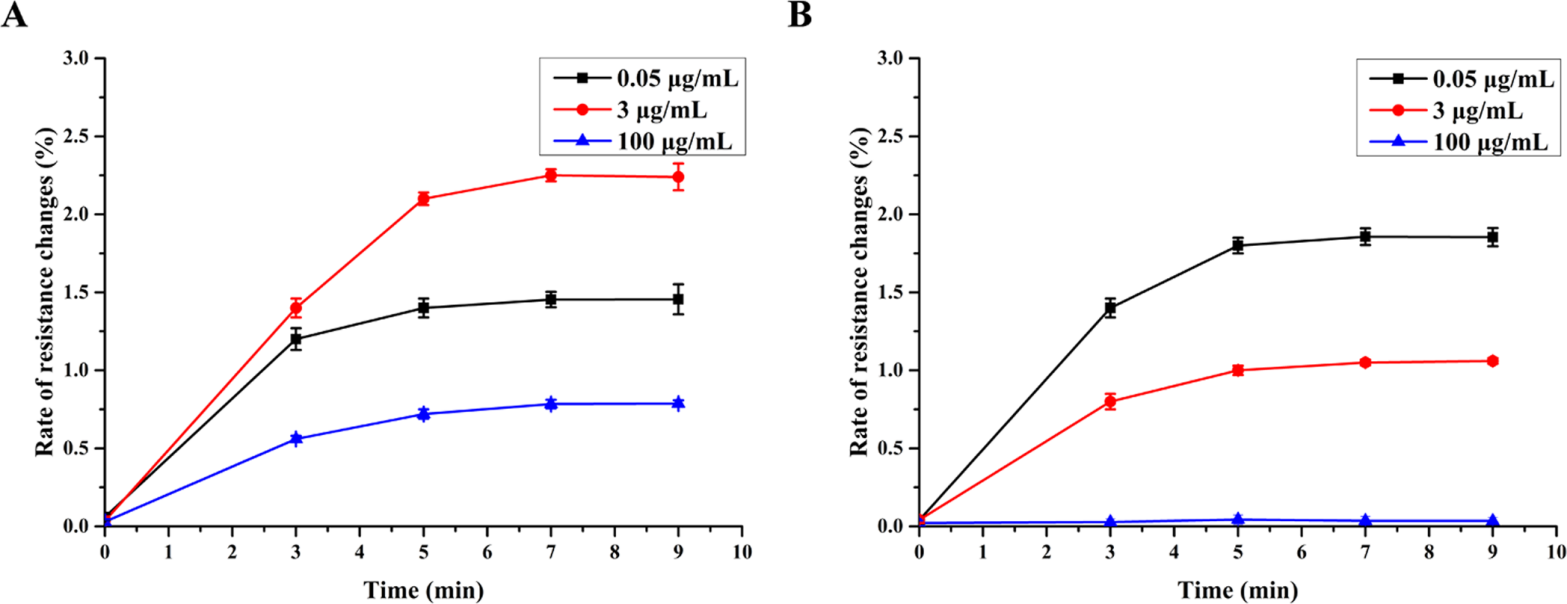
Optimization of the reaction time (*n* = 4). (A) Sandwich assay (sensor I). (B) Competitive assay (sensor II).

The reaction times for the binding of capture antibody to the detection antibody-bound CRP, and for the reaction of the remaining unreacted detection antibody to the sensor surfaces, are in this assay key performance parameters (cf. Figure 2B,C). Figure 5 shows for the optimized conditions of antibody and antigen concentrations, established as described above, the influence of the two reaction times on the sensor signals for the sandwich assay (Figure 5A) and the competitive assay (Figure 5B). The CRP concentrations of 0.05 (black lines), 3 (red lines), and 100 μg/mL (blue lines) were investigated. We established that at *t* = 7 min, both sensor responses reached a maximum, and used this as reaction time for the measurements.

A time for particle binding (cf. Figure 2D) of 6 min was used without further optimization.^23^ Furthermore, a 1 min washing step (50 μL total volume) prior to MNP binding was applied to remove unreacted assay components, and a 1 min washing step (50 μL total volume) after MNP binding was applied to remove the unbinding MNP.

### 3.2 Precision and Long-Term Stability and of the Assay

The numerical precision values of the measurements for a population *n* = 80, split into two concentration levels, are presented in Table 1. The measurement period is 20 consecutive days with two measurements per day and concentration level. The assay cartridges used were newly fabricated on the day of the measurement. The measurements on the same day are denominated as intra-assay, all measurements as interassay.

**Table 1:** Precision of the Assay (*n* = 80)

		intra-assay precision		interassay precision	
conc. (μg/mL)	mean (μg/mL)	SD (μg/mL)	RSD (%)	SD (μg/mL)	RSD (%)
5	5.012	0.437	8.71	0.465	9.28
100	99.41	8.485	8.54	7.359	7.40

We have also investigated the long-term stability of the reagent-loaded cartridge by incubating it at 37 °C over a 7-day period (Table 2). The recovery (94.2%–100.23) and relative standard deviation (RSD) < 10% (*n* = 6) obtained from this accelerated aging test suggest that the assay can maintain function for several months at 4 °C.

**Table 2:** Long-Term Stability of the Assay (*n* = 6)

time (d)	0		3		5		7	
conc. (μg/mL)	5	100	5	100	5	100	5	100
mean (μg/mL)	5.10	99.45	5.07	102.3	4.98	98.87	4.71	95.11
recovery (%)	102.0	99.5	101.4	102.3	99.6	98.9	94.2	95.1
SD (μg/mL)	0.43	7.12	0.30	9.35	0.36	5.86	0.28	6.66
RSD (%)	8.36	7.16	5.96	9.13	7.22	5.93	5.91	7.00

### 3.3 Establishment of Standard Curves

**Figure 6:**
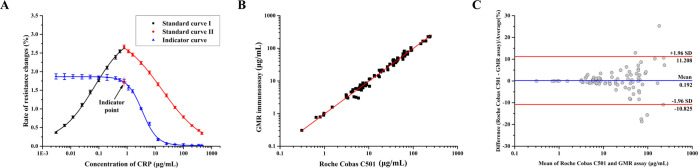
(A) Standard curves of the one-step sandwich assay combined with a competitive immunoassay for CRP detection (*n* = 4). (B, C) Comparison between the sensor and a commercial reference assay: the fitness analysis (B) and the Bland–Altman analysis for the validation of the GMR assay against the Roche Cobas C501 assay (C).

All standard curves were fitted by the four-parameter logistic model (*y* = *A*_2_ + (*A*_1_ – *A*_2_)/(1 + (*x*/*x*_0_)*^p^*)) (Figure 6A). For standard curve I (black line), *A*_1_ = 0.11964, *A*_2_ = 3.05252, *x*_0_ = 0.06783, *p* = 0.73045, *R*^2^ = 0.9988. For standard curve II (red line), *A*_1_ = 3.0816, *A*_2_ = −0.04743, *x*_0_ = 17.27685, *p* = 0.58217, *R*^2^ = 0.9998. For the indicator curve (blue line), *A*_1_ = 1.86524, *A*_2_ = 0.02144, *x*_0_ = 3.43843, *p* = 1.64857, *R*^2^ = 0.9995.

The three curves were then used for the quantification of CRP in the patient samples. Under the applied conditions, the “Hook” effect began to occur on sensor I (sandwich assay) at a CRP concentration of 0.781 μg/mL. The GMR signal on sensor II (competitive assay) was measured at that point as 1.745%, which was accordingly set as the indicator point of the assay.

### 3.4 Measurement and Validation

Validation of the measurements obtained with the GMR sensor device was performed against the Roche Cobas C501 optical assay (Figure 6B,C). Fitness analysis (*n* = 91) resulted in the following parameters for the data set, covering the concentration range between 3 and 350 μg/mL: *y* = 1.003*x* – 0.0104; *r* = 0.9881 (Figure 6B). In Figure 6C, the Bland–Altman plot of the relative differences between the data sets of the two compared assays is displayed. The mean relative difference is 1.96% (−10.825–11.208%, 95% confidence limit). A statistically significant bias between the two assays is not present.

## 4 Conclusions

We have developed and characterized the analytical performance of a tandem GMR sensor immunoassay for the biomarker CRP in human blood, featuring two different formats in an automated microfluidic sample handling cartridge. The combination enables measurement over the full clinically relevant concentration range. This means that the dilution of blood plasma samples is not required, which opens new possibilities for one-shot multimarker detection without compromising on low-abundance biomarkers.

Differential antibody/antigen coating of the individual GMR sensors was achieved by surface modification using the functional polymer PS-g-MAH, using a combination of spin-coating (polymer) and nanoplotting (proteins). The availability of the grafting polymer samples for research purposes is unfortunately still subject to a request to the manufacturer since the material is currently only commercially produced in industrial quantities.

The detection of the hook curve feature is easily applicable to a variety of different antibodies, giving the new assay concept a wide application scope, particularly for the POC diagnostics. This is supported by the assay’s low cost, short measurement time of ∼15 min, small sample size (50 μL), long shelf-life, and simple operation, which are all additional benefits. We successfully validated the tandem sensor assay against a commercial system, which established that the method has comparable figures of merit (e.g., limit of detection (LOD), LOQ) and would therefore be suitable for application in clinical testing. Reuse approaches could be investigated to satisfy sustainability demands, requiring a careful analysis of possible cross-contamination.
